# Inflammation as a Target in Cancer Therapy

**DOI:** 10.1155/2019/1971698

**Published:** 2019-03-07

**Authors:** Sonia Leon-Cabrera, Kathryn L. Schwertfeger, Luis I. Terrazas

**Affiliations:** ^1^Unidad de Biomedicina. Facultad de Estudios Superiores-Iztacala, Universidad Nacional Autónoma de México (UNAM), Av. De los Barrios 1, Los Reyes Iztacala, Tlalnepantla, Edo. De México 54090, Mexico; ^2^Carrera de Médico Cirujano, Facultad de Estudios Superiores Iztacala, UNAM, Av. De los Barrios Los Reyes Iztacala, Tlalnepantla, Edo. De México 54090, Mexico; ^3^Department of Lab Medicine and Pathology and Masonic Cancer Center, University of Minnesota, Minneapolis, Minnesota, USA

There is a strong association between cancer and inflammation. Dysregulated inflammatory responses play a pivotal role in tumor initiation, promotion, and progression through different pathways. Epidemiological evidence suggested that a stage of chronic inflammation, due to persistent infections like either parasites or viruses, or sterile inflammation associated with ambient factors are linked with tumorigenesis. Over the past decade, pharmacological inhibition of inflammatory cells and their products, together with the manipulation of genes involved in their functions, has been shown to participate in tumor incidence and progression. In consequence, cancer-promoting inflammation is an encouraging target of therapy in oncology. The list of tumor-promoting inflammatory cells includes tumor-associated macrophages (TAMs), dendritic cells, neutrophils, immature myeloid cells, mast cells, eosinophils, and lymphocytes. These cells are present at the tumor microenvironment and produce a variety of cytotoxic and inflammatory mediators, thus sustaining immunosuppression, tumor cell proliferation and survival, angiogenesis, autophagy, extracellular matrix breakdown, metastasis, chemoresistance, and radioresistance. Thus, understanding how inflammation in the whole tumor microenvironment can be targeted in more effective ways will ultimately lead to the development of therapeutic approaches that result in durable antitumor responses.

This special issue is aimed at encouraging the persistent effort to understand how the complex network of inflammatory circuits in the tumor microenvironment could be used to inform the development of new therapeutic modalities.

Within the tumor microenvironment, multiple mediators are secreted that contribute to the recruitment of circulating monocytes and the promotion of their differentiation into tumor-associated macrophages. Macrophages are important actors in the production of mediators and cytokines that favors inflammation, but on the other hand, could participate in wound healing and angiogenesis providing favorable conditions for tumor development. Thus, understanding the agents that are able to adjust the tumor microenvironment can be an effective way to obtain durable antitumor responses. In a research study by C. Hsieh and C.-H. Wang using an *in vitro* approach, the authors have demonstrated that aspirin inhibited the secretion of MCP-1, IL-6, and TGF-*β* by 4T1 breast cancer cells and regulated the expression of angiogenic and inflammation-associated cytokines in both malignant cells and macrophages. The authors postulate that aspirin increased M1 and decreased M2 polarization in macrophages, resulting in the restriction of communication in this microenvironment and reduced tumor progression.

Despite advances in understanding how inflammatory processes are involved in the development of melanoma and nonmelanoma skin cancer, surgical treatment is the gold standard therapy for basal cell carcinoma. V. Voiculescu et al. reviewed the mechanisms involved in topical therapies targeting the inflammation processes occurring in cutaneous carcinogenesis as an alternative to nonsurgical treatment. They discussed the mechanism involved in therapies targeting Toll-like receptor-7 (TLR-7) and showing that in association with radiotherapy, chemotherapy or immunotherapy have shown a superior success rate than monotherapy with minimal adverse reactions.

During esophageal carcinoma, local infiltration of inflammatory cells favors the interruption and deletion of the local basement membrane in esophageal squamous cells, favoring cell proliferation and the activation of nuclear factor kappa B (NF-*κ*B). The aberrant NF-*κ*B pathway is involved in the initiation and development of many malignant tumors and regulates transcription of target genes that control cell proliferation, apoptosis, invasion, and metastasis. Therefore, the inhibition of NF-*κ*B signaling could be an effective treatment against cancer and it could also restore sensitivity to other therapeutic options. F. Guo et al., in their experimental study, demonstrated that Grape seed proanthocyanidin (GSPE) extract inhibited the proliferation, induced apoptosis, and reduced the secretion of inflammatory cytokines in the human esophageal squamous cancer cell line. The authors postulated that GSPE activated caspase-3 and attenuated the activation of the NF-*κ*B signaling pathway by inhibiting the phosphorylation of I*κ*B, which could provide a potential new avenue for targeting this key pathway.

Autophagy is a regulated mechanism of the cell responsible for a self-degradative process, important for balancing sources of energy and maintaining metabolic homeostasis. Before the appearance of a tumor, autophagy helps in the degradation of damaged mitochondria that could otherwise induce oxidative stress, DNA damage, and genomic instability. In cancer, autophagy is a pathway used by tumor cells for recycling intracellular constituents, used as alternative energy sources during stressing conditions like hypoxia or nutrient deprivation. I. Cotzomi-Ortega et al. reviewed recent evidence about the interaction of autophagy with protein secretion pathways during carcinogenesis. They discussed the importance of establishing how autophagy regulates secretion from cancer cells depending upon cancer type or cancer stage, which could have implications in the use of autophagy inhibitors during clinical trials. I. Cotzomi-Ortega et al. proposed that manipulation of autophagy during cancer therapy should be used with caution since it could potentially promote malignancy and have other undesirable consequences.

CD44 is a cell surface glycoprotein mainly expressed in normal epithelial cells, and it has been proposed as a stem cell marker during tumorigenesis. By alternative splicing, human CD44 gene produces different CD44 isoforms expressed in different tissues during normal or disease stages. N. Suwannakul et al. evaluated the expression of CD44 variant 9 (CD44v9) in the liver of cholangiocarcinoma patients. They reported overexpression of CD44v9 and inflammation-related markers, in tissues from human liver fluke *Opisthorchis viverrini*-related cholangiocarcinoma. As cholangiocarcinoma is a chronic inflammation-induced cancer, they proposed that CD44v9 could be a biomarker for cancer stem cells in the progression of inflammation-related cholangiocarcinoma.

In summary, we are optimistic that the original research and review articles presented in this special issue will enhance the knowledge about the importance to understand how inflammatory pathways and mechanisms that regulate inflammation could lead to the development of better and novel biomarkers and therapies for cancer.

